# The Effects of Institution-Driven Entrepreneurial Education in Chinese Universities: A Qualitative Comparative Analysis Approach

**DOI:** 10.3389/fpsyg.2021.719476

**Published:** 2021-12-22

**Authors:** Rongzhi Liu, Yuxin Huo, Jing He, Dun Zuo, Zhiqiang Qiu, Jun Zhao

**Affiliations:** ^1^School of Business Administration, Zhongnan University of Economics and Law, Wuhan, China; ^2^Xinyu High-Tech Development Zone Tax Bureau, Xinyu, China; ^3^School of Public Administration, Zhongnan University of Economics and Law, Wuhan, China

**Keywords:** entrepreneurial education, China, QCA, curriculum system, teaching team

## Abstract

**Purpose:** This study aims to explore the effects of entrepreneurship education by examining the influences of the curriculum system, teaching team, design of practical programs, and the institutional systems on universities’ entrepreneurial education performance.

**Design/Methodology/Approach:** This paper employs a case-based approach—Qualitative Comparative Analysis (QCA). Data were collected from 12 universities that were typical cases in the implementation of entrepreneurial education. The four dimensions of entrepreneurship education are applied as conditional indicators. fsQCA3.0 software is used to analyze the necessary conditions and condition combination of the truth table.

**Findings:** There are three sets of condition combinations of the intermediate solution that results in a high level of entrepreneurial education performance: (1) when the credit ratio of entrepreneurship courses is higher and there are more practical platform platforms, even if the entrepreneurship education system and mechanism is less mature, the level of entrepreneurial education performance is high; (2) with a higher credit ratio of entrepreneurship courses, higher quality of teaching teams, and higher standard of practical platforms, the level of entrepreneurial education performance is high; (3) with a higher level of credit ratio of entrepreneurship courses and more practical platforms, as well as mature entrepreneurship education system and mechanism, even if the quality of the teaching team is lower, the level of entrepreneurial education performance is satisfied.

**Research Limitations/Implications:** The dimensions of entrepreneurship education can be expanded; additionally, given that there are many other factors affecting entrepreneurial performance, it is necessary to identify and integrate other possible factors on an ongoing basis.

**Practical Implications:** This study offers practical implications for universities and policy makers that can promote the transformation of theoretical knowledge into practice in the field of entrepreneurship in colleges and universities.

**Social Implications:** This study is one of the first to empirically examine the effect of institutional-driven entrepreneurship education in developing countries. The enhancement of entrepreneurship education can benefit the development of individuals and schools, and even has a potential impact on the progress of the country and society as a whole.

**Originality/Value:** This study emphasizes the significance of viewing the entrepreneurial education as a multi-dimensional concept by targeting different kinds of players. Furthermore, it employs a case-based approach to identify configurations of the antecedent attributes of the curriculum system, teaching team, design of practical programs, and the institutional systems, and their influence on universities’ entrepreneurial education performance.

## Introduction

Scholars have long been interested in the effect of entrepreneurial education on the potential entrepreneurs among university students (Sherman, 2005; Cruz et al., 2009; Li and Liu, 2011; Johansen, 2014). Entrepreneurship education has, therefore, emerged as a policy tool to stimulate entrepreneurial activities and encourage entrepreneurial intentions (Hoang, 2020; Zelin et al., 2021). Entrepreneurial education was first introduced into the higher education system in China in the 1990s, with the aim of enhancing potential entrepreneurs’ capability as well as their new venture creation performance. It is thought that the current achievements of entrepreneurial education can be summarized as follows: entrepreneurial education accelerates entrepreneurship, increases willingness to participate in entrepreneurship, develops entrepreneurial ability/skills, and influences entrepreneurship attitude (Martin et al., 2013; Nabi et al., 2017; Eesley and Lee, 2020; Jiatong et al., 2021). However, there are differences in evaluation standards, content definition, and methods set by different individuals in terms of the action mechanism; as a result, a clear and unified definition and complete measurement system for entrepreneurial education performance are lacking. Some studies have focused on the notion that entrepreneurial education enhances the positive perception of entrepreneurship, such as entrepreneurial intentions (Liñán et al., 2011), entrepreneurial self-efficacy, attitudes to entrepreneurship (Liu et al., 2019), and start-up behavior (Karlsson and Moberg, 2013). However, some studies on entrepreneurial education effects show contrasting results wherein the effect on students’ self-assessed entrepreneurial skills is insignificant and the effect on the intention to become an entrepreneur is even significantly negative (Hessel et al., 2008; Oosterbeek et al., 2010). Souitaris et al. (2007) found that entrepreneurial education had no significant effect on students’ perceived ability to become entrepreneurs. Fayolle et al. (2015) reported that the effects of an entrepreneurial education program are related to previous entrepreneurial exposure. Specifically, the positive effects are even more pronounced when previous enterprise exposures are weak or non-existent. In contrast, the results highlight significant negative effects of entrepreneurial education programs on students with previous experience of entrepreneurship.

Entrepreneurial education is a multi-dimensional concept; however, most of these studies disregard the fact that students tend to perceive their universities’ entrepreneurial education activities in a multidimensional rather than an one-dimensional way because they target different kinds of players. Aiming to determine the combinations of entrepreneurial education factors that affect new venture performance, this paper employs a case-based approach—Qualitative Comparative Analysis (QCA)— in order to identify configurations of antecedent attributes of the curriculum system, teaching team, design of practical programs, and the institutional systems, and their influence on universities’ entrepreneurial education performance.

Wu and Wu (2008) argued that the topic explored in this study has been well researched in the Western context but remains under-researched in the Asian-Pacific literature. In particular, in China, unlike the private-owned universities in the Western context, most of the universities are governed by the Ministry of Education; as a result, the evaluation of the effect of entrepreneurial education might be quite different. Some scholars have put forward multiple evaluation approaches, one of these involves assessing the effect of entrepreneurial education and training on the external economy, such as the probability, scale, and growth potential of college students to establish new enterprises; another approach is to evaluate the success of entrepreneurs, such as job satisfaction and personal income; and the third is to evaluate the range of entrepreneurial education, such as the number of participants, social recognition, economic benefits, and other aspects (Zheng et al., 2018). Research has focused on the direct and macro-level impact of entrepreneurial education; however, few studies examine how entrepreneurial education promotes the transformation of entrepreneurial performance and the mechanism of action involved (Walter et al., 2013).

Based on the above analysis, this paper aims to explore the effects of entrepreneurship education by examining the influences of the curriculum system, teaching team, design of practical programs, and the institutional systems on universities’ entrepreneurial education performance. The paper is structured as follows.

## Theoretical Background

### Curriculum Systems in Entrepreneurial Education

In order to realize the goal of cultivating the innovative and entrepreneurial spirit and consciousness among contemporary college students, improving their entrepreneurial ability, and enhancing their competitive strength to adapt to social development, colleges and universities conduct entrepreneurial education through the curriculum system (Kuratko, 2010; Rauch and Hulsink, 2015). As research expands, entrepreneurship education is expected to undergo some fundamental changes: it will see new concepts and course approaches being tested in the classroom that are very different from the past. The interaction between research, curriculum development, and teaching in the context of entrepreneurship is a close one (Ronstadt, 2017). Hao (2017) believes that the implementation of entrepreneurial education is a requirement for China’s higher education, and the reform of higher education must be realized through the construction of the curriculum system. The key to fostering the development of college students’ innovation and entrepreneurial education is to build a suitable curriculum of innovation and entrepreneurial education. In particular, a curriculum system suitable for professional development is needed for personnel training goals as well as to improve the core competitiveness of the professional. To develop the right mindset within students, which is one of the most important factors for successful entrepreneurship, the inclusion of entrepreneurship in a student’s education and curriculum is essential (Network, 2009). Entrepreneurship courses are vital components of a business school curriculum (Edelman et al., 2008). College entrepreneurial education can be divided into two formats, namely theoretical teaching and practical operations, according to the mode of teaching (Pittaway and Edwards, 2012). Therefore, from the perspective of form and content, entrepreneurial education can be classified under the entrepreneurship curriculum system and entrepreneurship practical education. The entrepreneurship curriculum system is the traditional form of face-to-face teaching between teachers and students, which enables direct learning. Based on the curriculum setting of college student training systems, entrepreneurship courses can be divided into compulsory courses, general courses, and professional courses. Some scholars believe that giving priority to the creation of core courses can enable a relatively solid foundation to be built for the curriculum system of entrepreneurial education, and supplementary courses can then be added according to the specific situation of the school and its development needs. Nabi et al. (2017) believe that the type of course represents a boundary condition that is worth exploring in order to obtain a more nuanced understanding of the impact of entrepreneurial education. The positive effect on perceived entrepreneurial skills could be upward-biased by the elective nature of the course (Rauch and Hulsink, 2015). Entrepreneurship course education is an essential element of entrepreneurial education. The course content will directly affect students’ understanding of innovation and entrepreneurship, and even their entrepreneurial intention. Karimi et al. (2015) found that elective entrepreneurial education programs significantly increased students’ entrepreneurial intention, although this increase was not significant for compulsory entrepreneurial education programs. Setting up entrepreneurship major courses can enable a focus on cultivating students who have strong innovation and entrepreneurship intention and possess certain entrepreneurial basic conditions that allow them to further master entrepreneurial skills (Letsoalo and Nguza-Mduba, 2020). Rauch and Hulsink (2015) believed that students attending electives are usually those who are interested in entrepreneurship and wish to acquire the skills necessary to become an entrepreneur, while entrepreneurial education in compulsory courses mainly teaches participants what entrepreneurship is. Therefore, the quality and content of entrepreneurial education curriculum should be targeted (Li-Qing and Yan-Qun, 2012), and the quantity should be of a certain scale, forming a relatively complete and reasonably structured curriculum system so as to have obvious guiding significance for students’ entrepreneurial intention and entrepreneurial activities (Hai-Zong and Yu-Dan, 2010).

#### Proposition 1

College students’ entrepreneurship performance is related to the curriculum system of entrepreneurial education.

### Teaching Team of Entrepreneurial Education

The professional teaching team in entrepreneurial education is a crucial factor for achieving the goal of an entrepreneurial education program (Seikkula-Leino et al., 2008; Teerijoki and Murdock, 2014; Ruskovaara and Pihkala, 2015). Some studies show that the professional background of teachers is related to the level and content of entrepreneurial education (Birdthistle et al., 2007; Draycott and Rae, 2011). According to Ruskovaara and Pihkala (2013), there is a connection between teachers’ cognition of their own entrepreneurial education skills and training and the implementation of entrepreneurial education. Studies have found that exemplary teachers increase students’ entrepreneurial intentions and improve their relevant attitudes and abilities (San-Martín et al., 2019). It is extremely important to construct a progressive entrepreneurial education system and allocate qualified teachers according to the level and structure of entrepreneurial education in order to promote entrepreneurial education (Wei and Guo, 2010). Seeking to improve the quality of entrepreneurial education, Huang et al. (2020) studied the factors that affect teachers’ competencies and proposed that in order to improve the quality of entrepreneurial education, it was necessary to adopt a new teaching model, pay attention to teacher career development, and improve the teacher evaluation and recruitment system. However, in entrepreneurial education, there are few studies on the influence of teachers and their backgrounds (Ruskovaara and Pihkala, 2015; San-Martín et al., 2019). For example, teachers of entrepreneurial education are mainly from the following backgrounds: first, full-time teachers specializing in entrepreneurial education teaching, mainly focusing on economic management, who have rich teaching experience and a solid theoretical knowledge foundation. Some teachers even serve as part-time consultants in enterprises and have close contact with the social economy. Second, there are part-time teachers encourage and guide students to invest in innovation and entrepreneurship ideologically, and help them to consult entrepreneurship related issues in the process. Third, entrepreneurs, venture capitalists, and other part-time teachers in off-campus enterprises with practical entrepreneurial experience who have practical entrepreneurial experience that full-time teachers lack, and can share the most direct entrepreneurial information and experience to students at the micro level. Fourth, part-time teachers who are experts from government departments, industrial and commercial finance experts, social organization scholars and others who have a macro-level understanding of entrepreneurship and the economy can predict and grasp the current entrepreneurial environment from a macro perspective to help avoid risks.

These teachers play an indispensable role in the process of entrepreneurial education in colleges and universities. This is the reason why we decided to consider the impact of entrepreneurial education on entrepreneurship performance through the teaching team.

#### Proposition 2

College students’ entrepreneurship performance is related to the teaching team.

### The Practical Platform

In terms of entrepreneurship practical platforms, the extracurricular activities in entrepreneurial education are complementary to classroom activities and the extracurricular practical activities in entrepreneurial education (Winkel et al., 2013), such as business competitions, networking events, guest talks, and student-led clubs, are increasing (Rae et al., 2012; Pittaway et al., 2015). Colleges and universities build various entrepreneurship practice bases and project incubation centers within the school to exercise and cultivate students’ comprehensive entrepreneurial quality and practical ability (Bonesso et al., 2018), to develop innovation and entrepreneurship ability, and provide a high quality of dual training (Hongtao and Junru, 2019). As a bridge between the campus and society, entrepreneurial practice can greatly help improve students’ entrepreneurial skills and entrepreneurial skills. Nabi et al. (2017) focused on what extracurricular activities are most effective in improving students’ entrepreneurial awareness, ability, and intention. Because of the practical nature of entrepreneurship, experiential learning opportunities are considered particularly important to facilitate the entrepreneurial learning process (Honig and Hopp, 2019). Various entrepreneurial practice platforms, such as incubation centers and entrepreneurial training bases at universities, can provide abundant practical experience and resources for college students, and greatly improve their skills and practical entrepreneurship abilities (Li and Ding, 2016; Gobi and Kumaran, 2020). The construction of entrepreneurship platforms needs support from various sources. Numerous colleges and universities that attach importance to the construction of these platforms invest a large amounts of human and financial resources into establishing them. The number of such colleges and universities can, to some extent, reflect the importance the universities attach to entrepreneurial education.

#### Proposition 3

College students’ entrepreneurship performance is related to the establishment of entrepreneurship practice platforms.

### Institutional System and Mechanism

Entrepreneurial education is a systematic project, involving educational administration, youth League committees, students, and other parties, and the division of labor between various school leaders. The development of entrepreneurial education requires the effective integration of resources within the school and the aggregation of resources outside the school so as to form a mutually helpful support system, division of labor, and cooperation mechanism. Formal education systems, especially higher education systems, influence the entrepreneurial attitudes and behaviors of students and graduates (Nowak, 2016). In order to promote the development of innovation education, it is necessary to accurately grasp the scientific connotations of innovation and the entrepreneurial education system, construct the promotion mechanism of innovation and entrepreneurial education, and strengthen the construction of the mechanisms for the motivation, formation, guarantee, and feedback in the context of innovation and entrepreneurial education (Nanzhong, 2016). In order to promote the development of entrepreneurial education, the mechanism of entrepreneurial education should be strengthened, including the construction of internal and external mechanisms; in other words, the coordination mechanism between school departments and the construction of cooperation mechanisms among the government, schools, and enterprises should be bolstered. The ecosystem of entrepreneurial education has become the most important and effective enterprise community participation and knowledge transfer mechanism within the university-industry-government framework, which creates value for the society and regional economy and emphasizes cooperation among universities, industries, and governments to promote the construction of the ecosystem of entrepreneurial education (Belitski and Heron, 2017). Bischoff et al. (2018) emphasized the importance of stakeholder collaboration in the entrepreneurial ecosystem of higher education institutions. The coordinated development of local universities and regional social development entrepreneurship is an important trend relating to the connotative development of entrepreneurial education in China (Yong and Yanqiang, 2019). In terms of the construction of the administrative leadership system, colleges and universities can set up a leading group for entrepreneurial education. The main leaders are appointed as the group leader, and the members include principals of academic affairs office, student affairs office, and youth League Committee to jointly plan and formulate the work plan for entrepreneurial education and the student training system. In terms of construction of the academic guidance system, external experts and scholars are invited to set up an academic committee of entrepreneurial education with teachers in the school that is responsible for the training of entrepreneurial education teachers and consultation and guidance for curriculum design; furthermore, other supporting departments are set up to develop innovation and entrepreneurial education in the school.

The improvement and development of entrepreneurial education systems in colleges and universities relies on a stable Institutional system and mechanism ensured by the government department. Therefore, the relationship between entrepreneurial education and entrepreneurial performance is as below:

#### Proposition 4

College students’ entrepreneurship performance is related to the institutional system and mechanism of entrepreneurial education.

## Materials and Methods

### The Qualitative Comparative Analysis Method

Qualitative comparative analysis (QCA), based on set and Boolean algebra, can integrate the advantages of qualitative and quantitative research methods and analyze the combination of antecedents leading to consequences.

The QCA method is used to study the impact of entrepreneurship education on entrepreneurial performance in colleges and universities, mainly based on the following considerations: first, the impact of entrepreneurship education on entrepreneurial performance of college students is not a simple process, and the interaction between the performance and development of entrepreneurship education in different dimensions will lead to different results. The level of entrepreneurship education in colleges and universities cannot be determined by a single factor, nor can it be determined by multiple factors respectively. Therefore, it is necessary to discuss multi-channel interaction from a holistic perspective. Secondly, when selecting the research sample of this paper, the universities with systematic entrepreneurship education and distinctive characteristics are not standardized, and the empirical requirements of large sample statistics cannot be met due to the specific data available. It is difficult to obtain the results of the document through regression analysis. QCA is a case-oriented research method. Each case is a complicated entity. During the analysis, the integrity of the case can be maintained and different parts of different cases can be linked together to depict the case as a combination of variables. Furthermore, this paper adopts clear set analysis to study the continuous variables representing different degrees.

### Sample Case Selection and Data Collection Process

This paper conducts research based on secondary data. Because of the large number of universities in China, the development level of the entrepreneurial education system is difficult to represent with complete data. If we choose to conduct an empirical investigation on the samples, controlling the number and scope of the respondents is challenging and the typicality and representativeness of the samples cannot be guaranteed. Second-hand data and QCA research on the basis of previous analysis can be used to accurately estimate the conditional variables. For the selection of cases, this paper mainly relies on the website of the Ministry of Education of China as well as various archives and official websites of universities, we selects 12 universities with prominent research on entrepreneurial education and pilot universities. In this paper, when the QCA method is used for analysis, the number of samples selected is 12 and the conditional variable is 4, which conforms to the number of explanatory variables (4∼7) in the interval of medium samples (10∼40). The samples selected in this paper are shown in Table 1.

**TABLE 1:** Universities’ data.

Serial number	University name	Colleges and universities type	Awards(Y)	Entrepreneurship course credits/total credits	Percentage of credits (mean value: 4.58%)	Curriculum system (R_1_)	Types of Faculty composition	Teaching team (R_2_)	Number of practice platforms (mean value: 8.58)	Practical education (R_3_)	the Institutional System and Mechanism (R_4_)
1	Renmin University of China	Subordinate 985	Inactive(0)	8/146	5.48%	Higher(1)	4	Stronger(1)	4	Less(0)	More mature(1)
2	Tsinghua University	Subordinate 985	Active(1)	6/141	4.26%	Lower(0)	4	Stronger(1)	10	More(1)	More mature(1)
3	Beijing University of Aeronautics and Astronautics	Subordinate 985	Active(1)	10/156	6.42%	Higher(1)	3	Weaker(0)	15	More(1)	Prematuration(0)
4	Shanghai Jiao Tong University	Subordinate 985	Active(1)	8/148	5.41%	Higher(1)	4	Stronger(1)	7	Less(0)	More mature(1)
5	Xi’an Jiaotong University	Subordinate 985	Active(1)	4/164	2.44%	Lower(0)	2	Weaker(0)	12	More(1)	Prematuration(0)
6	Wuhan University	Subordinate 985	Active(1)	8/150	5.33%	Higher(1)	4	Stronger(1)	14	More(1)	Prematuration(0)
7	Northwestern Polytechnical University	Subordinate 985	Active(1)	10/158	6.33%	Higher(1)	4	Stronger(1)	11	More(1)	Prematuration(0)
8	Heilongjiang University	provincial	Active(1)	4/160	2.50%	Lower(0)	2	Weaker(0)	4	Less(0)	More mature(1)
9	Wenzhou University	provincial	Inactive(0)	4/148	2.70%	Lower(0)	4	Stronger(1)	3	Less(0)	More mature(1)
10	Zhejiang University	Subordinate 985	Active(1)	9/152	5.92%	Higher(1)	4	Stronger(1)	12	More(1)	More mature(1)
11	Nanjing University of Finance and Economics	Provincial	Inactive(0)	12/165	7.27%	Higher(1)	3	Weaker(0)	8	Less(0)	Prematuration(0)
12	Northeast Normal University	Subordinate 211	Inactive(0)	5/149	3.36%	Lower(0)	4	Stronger(1)	3	Less(0)	More mature(1)

*Source: The website of the Ministry of Education of China, various archives and official websites of universities.*

### The Assignment Basis

The effect of entrepreneurial education on the entrepreneurial performance of college students is complex, and the interaction between performance and development of entrepreneurial education in various dimensions will lead to different results. The level of entrepreneurial education in colleges and universities cannot be determined by a single factor or by multiple factors separately. Therefore, it is necessary to discuss multi-dimensional interaction from a holistic perspective. This study examines the relationship between entrepreneurship performance and the following four factors of entrepreneurial education: curriculum system (R_1_), teaching team (R_2_), practical platform (R_3_), and the institutional system and mechanism (R_4_). These conditional indicators, namely R_1_, R_2_, R_3_, and R_4_, are shown in Table 2. When collecting data, this paper selects four senior students from each case University, investigates the total professional credits and the credits of entrepreneurship courses, and makes the average statistics of the proportion. The proportion of each case is compared with the mean value. If it is higher than the mean value, it will be assigned 1 to the relevant indicator R, and if it is lower than the mean value, it will be assigned 0.

**TABLE 2:** Definition of variables.

Variable	Indicators category	Index assignment criteria
Outcome variable: Y		If the sample universities have won two gold awards or above (first, second, and third place) in the 1st to 5th Internet + innovation and entrepreneurship competition for college students, they will show positive performance and assign 1; otherwise, they will be assigned 0.
Conditional indicators:		
Curriculum system (R_1_)	Continuous variable	If the credit proportion of entrepreneurial education courses is higher than the mean, the value is 1; if the credit proportion is lower than the mean, the value is 0.
Teaching team (R_2_)	Continuous variable	If the entrepreneurial teacher team consists of > 3, the value is 1; otherwise, it is 0
Practical education (R_3_)	Continuous variable	If the number of entrepreneurial practice platforms on campus is higher than the mean, the value is 1; if it is lower than the mean, the value is 0
Institutional mechanism (R_4_)	Categorical variable	An independent school of entrepreneurship is assigned a value of 1, otherwise 0

The assignment bases of R_1_, R_2_, R_3_, and R_4_ are as below:

(1) R1: According to Yuan Liping and Yang Yang’s visual analysis of the course research of entrepreneurship education in colleges and universities, to build a complete knowledge system of entrepreneurship, colleges and universities should try their best to ensure that students have enough course choices and relatively high-frequency courses to learn about entrepreneurship. At present, the total course credits of students in 4-year universities are between 120 and 180, and most of the courses related to entrepreneurship education are 2 credits. If the students’ course credits of a university take the median of 150 and the credits of entrepreneurship education courses reach 10 points (more than 5 courses), students have the conditions to participate in entrepreneurship knowledge learning for five semesters, and the proportion of entrepreneurship education courses is slightly higher than 6%.

(2) R2: The professional team of entrepreneurship teachers is a crucial factor to achieve the goal of entrepreneurship education. For example, entrepreneurship education teachers mainly come from: (a) Full-time teachers specializing in entrepreneurship education and teaching, (b) part-time teachers engaged in other professional work on campus but assisting students in learning entrepreneurship knowledge, (c) entrepreneurs, venture capitalists and other part-time teachers with practical entrepreneurship experience outside the school, (d) experts from government departments, industrial and commercial finance experts, Social organization scholars and other part-time teachers with macro understanding of entrepreneurship and economy. When there are more than three types of entrepreneurial teachers in colleges and universities, it indicates that the school has a variety of teacher resources both inside and outside the school, which to some extent can ensure that students can receive the joint guidance of theoretical and practical education from teachers.

(3) R3: The establishment of entrepreneurial practice platform in schools can cultivate college students’ entrepreneurial practical ability, which is a new way to carry out entrepreneurship education at present. Various entrepreneurial practice platforms in colleges and universities (such as incubation centers and entrepreneurial training bases) can provide rich practical experience and available resources for college students, and greatly improve their practical entrepreneurial skills and levels. Entrepreneurial platform construction needs the support of various forces. Many colleges and universities that attach importance to platform construction have invested a lot of money and property to create it, and the amount of money can show the importance attached by the school to entrepreneurship education to a certain extent.

(4) R4: The improvement and development of entrepreneurship education system in colleges and universities need to rely on a stable system and mechanism. The independent entrepreneurship college can implement the responsibility subject, clearly assess Mubao, give full play to organizational advantages and platform functions with invisible college and tangible operation, achieve a high degree of integration of resources inside and outside the school, further promote the continuous renewal of innovative and efficient entrepreneurship education concept, and implement practical education and innovative development.

The outcome variable Y in this paper is based on whether the sample universities have won two gold awards or above (first, second, and third place) in the 1st to 5th Internet + innovation and entrepreneurship competition for college students. If they have won, they will show positive performance and be assigned 1; otherwise, they will be assigned 0. The universities that often participate and win awards have a positive correlation with the level of entrepreneurial education (Ren et al., 2017). Entrepreneurship competition and college students’ entrepreneurship and entrepreneurial ability strong correlation, and Internet + contest of college students’ innovative undertaking led by the Ministry of Education of China held be influential and appeal, the game be held once a year, college students before and after the entry has a year to accept entrepreneurial education and practice, from the time interval is conform to the law of education (Yan et al., 2018). Entrepreneurial performance is mainly based on the financial status of college students’ entrepreneurial enterprises, which has strong influence on the characteristics of individual entrepreneurship. However, the research sample selected in this paper comprises college groups, whose awards in entrepreneurship competitions reflect the effect and level of entrepreneurial education and teaching activities of this school to a certain extent. In the Internet + Innovation and Entrepreneurship Competition for college students, the gold award or above (first, second, or third) projects attract the attention of the outside world and the investors, or lead to financing to transfer equity, or increase the success rate of applications for academic patents. Therefore, this paper adopts the competition award to measure the performance of the university’s entrepreneurial education. See Table 1 for details.

## Results and Discussion

In this paper, it is shown that entrepreneurial education, being a systematic concept, has many dimensions that will affect entrepreneurship performance. Therefore, five factors were analyzed and the awards of universities in the Internet + Entrepreneurship competition were taken as outcome variables. The curriculum system (R_1_), teaching team (R_2_), practical platform (R_3_), and the institutional system and mechanism (R_4_) were used as conditional indicators.

Thus, all variables are assigned values. Based on qualitative comparison and analysis, this paper summarizes the assignment values of different conditional indicators of each sample and combines data with result variables to obtain a truth table (see Table 3).

**TABLE 3:** Table of truth.

The case number	R_1_	R_2_	R_3_	R_4_	Y
1	1	1	0	1	0
2	0	1	1	1	1
3	1	0	1	0	1
4	1	1	0	1	1
5	0	0	1	0	1
6	1	1	1	0	1
7	1	1	1	0	1
8	0	0	0	1	1
9	0	1	0	1	0
10	1	1	1	1	1
11	1	0	0	0	0
12	0	1	0	1	0

*R1, curriculum system; R2, teaching team; R3, practical platform; R4, the institutional system and mechanism.*

### Sufficiency Analysis

Consistency and coverage are two key indicators in QCA to determine the degree of correlation between the conditional combination and outcome variables. According to the QCA method, 0.8 is generally regarded as the peak of consistency. For instance, when the consistency of the variable combination A*B*C is higher than 0.8, the combination can be regarded as an interpretation of the result variable. For coverage, it represents the explanatory strength of the variable combination, which is proportional to the value. The truth table is input into the QCA software and operated stepwise to obtain the truth analysis table, as shown in Table 4 (complex solution result), Table 5 (simple solution result), and Table 6 (intermediate solution result).

**TABLE 4:** fsQCA output: complex solution result.

Complex solution result			
**Condition combination**	**Original coverage**	**Net coverage**	**Consistency**
R_2_*R_3_*∼R_4_	0.5	0.25	0.888889
R_1_*R_2_*R_3_	0.5	0.25	1
R_1_*∼R_2_*∼R_3_*R_4_	0.125	0.125	1
Result coverage	0.875		
Result consistency	0.93333		

*Source: The Authors’.*

*Means the variable combination.

**TABLE 5:** fsQCA output: simple solution result.

Simple solution result			
**Condition combination**	**Original coverage**	**Net coverage**	**Consistency**
R_1_	0.875	0.875	0.777778
∼R_2_*∼R_3_	0.125	0	0.666667
∼R_1_*∼R_2_	0.125	0	1
∼R_2_*R_4_	0.125	0	1
Result coverage	0.875		
Result consistency	0.777778		

*Means the variable combination.

**TABLE 6:** fsQCA output: intermediate solution result.

Intermediate solution result			
**Condition combination**	**Original coverage**	**Net coverage**	**Consistency**
R_1_*R_3_*∼R_4_	0.5	0.25	0.888889
R_1_*R_2_*R_3_	0.5	0.25	1
R_1_*∼R_2_*R_3_*R_4_	0.125	0.125	1
Result coverage	0.875		
Result consistency	0.93333		

*Means the variable combination, R1*R3*∼R4 = Higher credit ratio of entrepreneurship courses in colleges and universities *More practical platforms in colleges and universities *Less mature entrepreneurial education system and mechanism in colleges and universities, R1*R2*R3 = Higher credit ratio of entrepreneurship courses *Higher quality of teaching team *Higher practical platforms, R1*∼R2*R3*R4 = Higher credit ratio of entrepreneurship courses in colleges and universities *less competent or less skilled teachers *more practical platform platforms in colleges and universities *Mature entrepreneurial education system and mechanism in colleges and universities.

Through clear set detection, via necessity condition analysis and condition combination analysis, it can be shown that the three sets of condition combinations of intermediate solution results are all core conditions, which are expressed as follows:

${\text{Result = R}}_{\text{1}}{\text{*R}}_{\text{3}}\text{*}∼{\text{R}}_{\text{4}}{\text{ + R}}_{\text{1}}{\text{*R}}_{\text{2}}{\text{*R}}_{\text{3}}{\text{+R}}_{\text{1}}\text{*}∼{\text{R}}_{\text{2}}{\text{*R}}_{\text{3}}{\text{*R}}_{\text{4}}$

= Higher credit ratio of entrepreneurship courses in colleges and universities * More practical platforms in colleges and universities * Less mature entrepreneurial education system and mechanism in colleges and universities.

= Higher credit ratio of entrepreneurship courses * Higher quality of teaching team * Higher practical platforms.

= Higher credit ratio of entrepreneurship courses in colleges and universities * less competent or less skilled teachers * more practical platform platforms in colleges and universities * Mature entrepreneurial education system and mechanism in colleges and universities.

When curriculum education, faculty, practical platform, and the institutional system and mechanism are the four dimensions of entrepreneurial education, entrepreneurial education can promote college students to achieve positive entrepreneurial performance in three ways.

(1) Higher credit ratio of entrepreneurship courses in colleges and universities * More practical platforms in colleges and universities * Entrepreneurial education systems and mechanisms in colleges and universities are not sufficiently mature.

For example, in the case of Wuhan University and Northwestern Polytechnical University, although these universities do not have a special independent entrepreneurial education college, but all plans for students a more entrepreneurial course credits for students to learn, can bring good implementation in the cultivation of students innovative undertaking system, objectively to ensure continuous term of entrepreneurial knowledge, guide the entrepreneurial education combined with professional education, optimizing the course system construction, and according to different students have better entrepreneurship training; furthermore, the school has multiple base of business practice, set up the innovative entrepreneurial development center, to carry out a variety of activities. It also plays a systemic role in laying a foundation for students to accumulate entrepreneurial experience, which, to a certain extent, compensates for the lack of a mature university system and mechanism.

(2) Higher credit ratio of entrepreneurship courses * Higher faculty * More practical platforms in colleges and universities.

The combination of conditions indicates that in entrepreneurial education, when colleges and universities offer a large number of credits innovation and entrepreneurship courses, have competent teachers, and possess multiple platforms to carry out entrepreneurial practice activities on campus, the development of entrepreneurial education in colleges and universities can have a positive impact on students’ entrepreneurial performance.

In the case of Zhejiang University, a typical research university, innovation and entrepreneurial education programs required more (elective) course credits for students in order to meet the needs of students to carry out innovation and entrepreneurship in various ways. The school attaches great importance to the enrichment and development of entrepreneurial education teachers. In addition to established full-time lecturers, it also employs off-campus entrepreneurs, government workers, investors, and other professionals as part-time teachers. Furthermore, it actively carries out diverse entrepreneurial practice activities, relies on school-enterprise cooperation, creates a constructive and free entrepreneurial atmosphere, sets up entrepreneurial training bases, and sets entrepreneurial goals. Zhejiang University takes a leading position in the construction and implementation of a holistic entrepreneurial education system, paying attention to the establishment of students’ innovative consciousness, the cultivation of innovative spirit, and the improvement of innovative ability, which promotes the achievement of practical entrepreneurial goals by students.

(3) Higher credit ratio of entrepreneurship courses in colleges and universities * Lower quality of teaching team * More practical platforms in colleges and universities * Mature entrepreneurial education system and mechanism in colleges and universities.

The combination of conditions indicates that, in undertaking entrepreneurial education, colleges and universities offer relatively high credits for innovation and entrepreneurship courses, have relatively weak faculty, and possess many entrepreneurial practice platforms. When the entrepreneurial education system and mechanism is mature, entrepreneurial education in colleges and universities can have a positive influence on students’ entrepreneurial performance.

Shanghai Jiao Tong University offers high-quality and high-volume entrepreneurial education courses and designs course design modules with the characteristics of university programs, realizes elite entrepreneurship training through different modules, and meets the needs of students in terms of credits. However, the composition of the faculty undertaking entrepreneurial education is not sufficiently diversified. The School of Entrepreneurship has been established, and all the students of the university can participate in entrepreneurship courses. Shanghai Jiao Tong University attaches importance to the curriculum theory, applied practice, effective entrepreneurship practical activities, and student entrepreneurial base through various practices to cultivate entrepreneurial talents and careers in order to attract more students to entrepreneurial practice, develop the school as a platform, attract the enterprise cooperation from outside of the universities, provide an incubation service for entrepreneurial teams.

### Necessity Analysis

A necessary condition is a variable that has a decisive influence on the result. For example, if A is a necessary condition for B, then A can be said to cause the result B. This study uses software fsQCA3.0 for calculations, and the results are shown in Table 7. In QCA, similar to the role of regression coefficients in regression analysis, a consistency index indicates whether a conditional variable that affects the outcome variable must exist when the result variable is assigned to 1. This condition variable is a necessary condition for the outcome variable only when the consistency index is 0.9 or greater. All conditional variables in the table fail to meet this level, indicating that Curriculum system, faculty, Practical platform, and Institutional System and Mechanisms are insufficient to constitute the necessary conditions for universities to win two or more gold awards in the Internet + Entrepreneurship competition. Therefore, the result variable Y needs the joint action of multiple factors, and a single condition cannot promote the emergence of the result variable Y.

**TABLE 7:** Necessity analysis.

Conditional indicator	Consistency	Coverage
R_1_	0.875000	0.777778
∼R_1_	0.125000	0.333333
R_2_	0.625000	0.625000
∼R_2_	0.375000	0.750000
R_3_	0.875000	0.933333
∼R_3_	0.125000	0.222222
R_4_	0.500000	0.571429
∼R_4_	0.500000	0.800000

## Conclusion

### Summary of the Research Findings

This paper uses the QCA method to assess the effect of entrepreneurship education on the entrepreneurial performance of college students in China, in order to verify the impact on entrepreneurship performance from different levels.

First, the promotion effect of entrepreneurship education on university entrepreneurship performance should be enhanced via multiple dimensions. This paper examines 12 universities and conducts QCA on the factors influencing the universities’ performance in an entrepreneurship competition from the perspective of Curriculum system, Teaching team, Practical platform, and institutional system and mechanism. From the data, these conditions are found to work together and interact. In the development of entrepreneurship education, colleges and universities should therefore give consideration to various factors.

In terms of Curriculum system, colleges and universities should actively incorporate double gen classes in their student training systems, in the general education curriculum, the setting of Creative Thinking Development (CTD), entrepreneurship practice, and employment guidance. Examples include compulsory courses of foundation classes, supplemented by entrepreneurial skills upgrading, and entrepreneurship. Different specialties are effective to cultivate students’ double gen spirit and consciousness, and help them learn to apply innovative learning in all aspects of life. Full use should be made of case studies, curriculum education reform should be carried out, and innovation and entrepreneurship education should be integrated into professional teaching courses.

In terms of teaching teams, colleges and universities should seek to constantly optimize the structure of the faculty engaged in entrepreneurship education and teaching activities, select teachers with strong innovative and entrepreneurial spirit and rich entrepreneurial experience both on and off-campus, and let teachers drive students to form an entrepreneurial team and participate in entrepreneurial practice, so that both teachers and students can gain practical experience. Colleges and universities should also keep in close contact with the wider society and invite outstanding entrepreneurs and venture capitalists from various industries to serve as part-time teachers at the school in order to promote the effective integration and transformation of students’ entrepreneurial theoretical knowledge into entrepreneurial practice and continuously strengthen school-enterprise cooperation.

In terms of practical platforms, colleges and universities should make full use of technical facilities in the business incubation spaces in order to speed up the integration of school and social resources, promote all kinds of scientific research at colleges and universities, improve the results of academic projects and the integration of students’ business needs, and constantly strive for collaboration with external parties such as the government, enterprises, social organizations, and other support systems for entrepreneurship practice of colleges and universities, enrich and strengthen the construction of campus entrepreneurial platforms, realize the entrepreneurial practice project on the ground and business incubation facilities. Through various media such as innovation and entrepreneurship competitions, university science and technology parks, and entrepreneurship training bases, entrepreneurship projects can be seamlessly connected, attracting various types of financing and investment, enabling substantive collaboration and cooperation, and sharing entrepreneurial achievements with the wider society.

In terms of the institutional system and mechanism, colleges and universities should seek to establish a systematic and scientific management system and a system of accountability for entrepreneurship education. Schools should establish systems for innovation and entrepreneurship administrative leadership, academic guidance, teaching implementation and other aspects, clear leadership responsibilities, personal accountability, and set up a special committee responsible for entrepreneurship education. In addition, schools should aim to set up a multilevel entrepreneurship education center, arrange entrepreneurship teaching by teaching teams, and implement the development of entrepreneurship education from a holistic perspective.

### Limitation and Future Research

There are some limitations in the selection of cases in the research and design of this paper. Although the selected sample universities are relatively leading in the development of entrepreneurship education in China, they can not represent the actual level of the whole university circle, so the number of cases needs to be increased in future research.

When determining the dimension of entrepreneurship education, this paper adopts the text analysis method, which is summarized from the main concerns of the entrepreneurial education policy text adopt by the ministry of education in China. Therefore, the dimensions of entrepreneurship education mentioned in this paper is limited in Chinese conditions, there should be more factors affecting entrepreneurial performance, and it is necessary to increase and integrate possible factors in future research.

## Data Availability Statement

The original contributions presented in the study are included in the article/supplementary material, further inquiries can be directed to the corresponding author/s.

## Author Contributions

RL and ZQ: conceptualization and resources. YH and DZ: methodology. JZ: formal analysis and investigation. DZ: writing—original draft preparation. RL and YH: writing—review and editing. RL, JZ, and ZQ: funding acquisition. RL and ZQ: resources. JH: revision. All authors contributed to the article and approved the submitted version.

## Conflict of Interest

The authors declare that the research was conducted in the absence of any commercial or financial relationships that could be construed as a potential conflict of interest.

## Publisher’s Note

All claims expressed in this article are solely those of the authors and do not necessarily represent those of their affiliated organizations, or those of the publisher, the editors and the reviewers. Any product that may be evaluated in this article, or claim that may be made by its manufacturer, is not guaranteed or endorsed by the publisher.
